# Age- and diet-dependent progression of retinal microvascular injury in GCK-MODY under metabolic stress

**DOI:** 10.3389/fendo.2026.1744691

**Published:** 2026-03-06

**Authors:** Yadi Huang, Yang Liu, Yiqing Wang, Xuan Liu, Shuhui Ji, Shanshan Chen, Hua Shu, Yu Fan, Ming Liu, Xin Li

**Affiliations:** 1 Department of Endocrinology and Metabolism, Tianjin Medical University General Hospital, Tianjin, China; 2 Henan Key Laboratory of Rare Diseases, Endocrinology and Metabolism Center, The First Affiliated Hospital, and College of Clinical Medicine of Henan University of Science and Technology, Luoyang, China

**Keywords:** apoptosis, diabetic retinopathy, GCK-MODY, inflammation, oxidative stress

## Abstract

**Background:**

Maturity-onset diabetes of the young type 2 (GCK-MODY), caused by heterozygous inactivating mutations in the glucokinase (GCK) gene, is generally considered a mild and stable form of diabetes with a relatively low risk of chronic complications. However, whether GCK deficiency predisposes to retinal microvascular injury under metabolic stress remains unclear.

**Methods:**

A GCK-Q26L knock-in mouse model (GCK^Mut^) was used to evaluate age- and diet-dependent alterations in retinal morphology and molecular pathology under normal diet (ND) and high-fat diet (HFD) conditions at 28, 40, and 60 weeks. Retinal structure and vasculature were examined by H&E staining and trypsin digestion. Oxidative stress, inflammation, and apoptosis were assessed using dihydroethidium fluorescence, Western blotting, and immunohistochemistry. Correlation analyses were performed to determine the relationship between NOX2 expression and inflammatory/apoptotic markers.

**Results:**

Under ND, retinal morphology and microvasculature were comparable between GCK^Mut^ and WT mice at 28, 40, and 60 weeks. In contrast, after prolonged HFD exposure, 60-week-old GCK^Mut^ mice exhibited clear microvascular injury, characterized by increased acellular capillaries and pronounced pericyte loss. At this late stage, retinal ROS levels were elevated, accompanied by NOX2 upregulation and increased expression of IL-1β and TNF-α. Apoptotic signaling was concurrently enhanced, as reflected by increased cleaved caspase-3 and a higher Bax/Bcl-2 ratio. Consistently, NOX2 protein levels correlated positively with inflammatory and apoptotic markers.

**Conclusions:**

This study demonstrates that GCK inactivation can predispose to retinal microvascular injury under prolonged metabolic stress. These findings support a NOX2-centered oxidative stress–linked inflammatory and apoptotic axis in late-stage retinal injury and highlight potential therapeutic targets for risk reappraisal in GCK-MODY.

## Introduction

Maturity-onset diabetes of the young type 2 (GCK-MODY) is caused by heterozygous inactivating mutations in the glucokinase (GCK) gene. Clinically, it presents with lifelong, stable, mild fasting hyperglycemia, and ketoacidosis or severe metabolic decompensation is uncommon (1–4). Glycemic levels typically remain steady over time and often do not require glucose-lowering therapy. Accordingly, GCK-MODY has traditionally been regarded as carrying a low risk of chronic microvascular complications (5–7). Our previous studies showed that GCK-inactivating mutant mice (GCK-Q26L) exhibit age- and diet-dependent alterations in lipid metabolism and renal microvascular pathology, suggesting that the metabolic stress sensitivity of GCK mutation carriers may be underestimated (8). In addition to the kidney, the retina is another densely perfused microvascular organ. However, its susceptibility to GCK mutations remains largely unexplored, particularly under chronic high-fat diet (HFD) conditions, where the underlying pathological mechanisms are poorly defined.

Diabetic retinopathy (DR) is a leading cause of adult-onset blindness worldwide (9–11). Early manifestations of DR include acellular capillary formation, pericyte loss, and capillary remodeling, which may progress to irreversible visual impairment, such as neovascularization and macular edema (12, 13). Oxidative stress induced by chronic hyperglycemia is a critical initiating factor in the pathogenesis of DR (14, 15). The accumulation of reactive oxygen species (ROS) can activate mitochondrial apoptotic pathways and promote the expression of proinflammatory cytokines, including IL-1β and TNF-α (16, 17). Among ROS-generating enzymes, the NADPH oxidase isoform NOX2 is a major source. It is broadly expressed in retinal glial and vascular endothelial cells and is persistently activated under hyperglycemic conditions (18, 19). Moreover, oxidative stress and inflammation not only directly contribute to retinal pathology but also accelerate DR progression by inducing apoptosis (20, 21). Although these signaling pathways have been extensively studied in type 1 and type 2 diabetes, their roles in atypical subtypes such as GCK-MODY remain poorly characterized.

In light of these observations, we employed a GCK-mutant mouse model (GCK^Mut^) to investigate the age-dependent progression of retinal structural and molecular pathology under both normal and high-fat diets. We systematically assessed dynamic changes in retinal microvascular structure, oxidative stress, inflammation, and apoptosis. Correlation analyses were performed to explore the potential role of NOX2 in the oxidative stress–inflammation–apoptosis signaling cascade. This study aims to elucidate the molecular mechanisms through which metabolic stress induces retinal injury in GCK-MODY. The findings may provide mechanistic insight and identify potential therapeutic targets for re-evaluating chronic complication risk in this diabetic subtype.

## Materials and methods

### Animal models

Heterozygous GCK-Q26L knock-in mice (GCK^Mut^) on a C57BL/6J background were bred in-house and used in this study (22). Wild-type C57BL/6J mice served as controls. The normal diet (ND; SCXK 2018-0003) comprised 20% protein, 4% fat, and 5% fiber, whereas the high-fat diet (HFD; D12492, Research Diets) contained 20% protein, 60% fat, and 20% carbohydrates. After weaning, male mice were divided into four groups according to genotype and diet: WT+ND, GCK^Mut^+ND, WT+HFD, and GCK^Mut^+HFD. All mice were maintained under specific pathogen-free (SPF) conditions with a 12-hour light/dark cycle at 22 ± 2 °C, with ad libitum access to food and water.

### Retinal morphological analysis

After anesthesia, mouse eyes were fixed in 4% paraformaldehyde, embedded in paraffin, and sectioned at 4 μm thickness. All sections were prepared in a consistent orientation and included the optic nerve head. Retinal sections were stained with hematoxylin and eosin (H&E), examined under a light microscope, and quantified at a fixed distance from the optic nerve head. Retinal thickness was measured using Image-Pro Plus 6.0 software.

### Retinal trypsin digestion

Retinal vascular flat mounts were prepared using a previously described trypsin digestion protocol (23–25). Briefly, retinas were isolated, washed, and digested with trypsin (G4004; Servicebio) to remove non-vascular tissue. The isolated vascular networks were subsequently mounted on glass slides. Slides were stained with periodic acid–Schiff (PAS) and hematoxylin. Images were acquired from 5–6 fields spanning the central to peripheral retina using a light microscope. Acellular capillaries and pericyte ghosts were quantified per square millimeter.

### Immunohistochemistry

After deparaffinization, antigen retrieval was performed, and endogenous peroxidase activity was blocked. Sections were incubated overnight at 4°C with an anti-cleaved caspase-3 primary antibody (A2156; ABclonal). An HRP-conjugated secondary antibody was applied, followed by DAB staining and hematoxylin counterstaining. Stained sections were imaged using a light microscope, and quantification was performed with Image-Pro Plus 6.0.

### Reactive oxygen species detection

Frozen retinal sections were incubated with dihydroethidium (DHE; R001, Vigorous Biotechnology) at 37°C for 30 minutes. After staining, images were promptly captured using a Zeiss AxioImager M2 fluorescence microscope. ROS fluorescence intensity was quantified using Image-Pro Plus 6.0.

### RNA extraction, cDNA synthesis, and quantitative real-time PCR

Total RNA was isolated from retinal tissues using TRIzol reagent (Invitrogen, USA) according to the manufacturer’s instructions. Reverse transcription was carried out with the PrimeScript™ RT kit with gDNA Eraser (Takara, Japan) to generate complementary DNA. Quantitative real-time PCR was performed on an ABI detection system using TB Green Premix Ex Taq™ (Tli RNaseH Plus) (Takara, Japan) in a final reaction volume of 20 μL. GAPDH was selected as the endogenous reference gene. All primer sequences were designed and synthesized by ZTSINGKE Biological Technology Co., Ltd. (Beijing, China), and are provided in **Supplementary Table 1**.

### Western blot analyses

Retinal proteins were extracted with RIPA buffer, separated by SDS-PAGE, and transferred onto nitrocellulose membranes. After blocking, membranes were incubated with primary antibodies targeting IL-1β (A16288; ABclonal), TNF-α (A11534; ABclonal), NOX2 (BM4576; BOSTER), SOD2 (PB9442; BOSTER), Bcl-2 (BA0412; BOSTER), Bax (A00183; BOSTER), Cleaved caspase-3 (A2156; ABclonal), p-S6K (AP0564; ABclonal), S6K (A4898; ABclonal), p-S6 (AP1328; ABclonal), S6 (A11874; ABclonal), and β-actin (KM9001T; Sungene). Protein bands were visualized by enhanced chemiluminescence (ECL) detection (CLiNX).

### Statistical analysis

All assays were performed with at least three technical replicates when applicable, and data are expressed as mean ± standard error of the mean (SEM). Unpaired t-tests were used for group comparisons, and linear regression was applied for correlation analysis. A p-value < 0.05 was considered statistically significant. All statistical analyses were performed using GraphPad Prism version 8.0.

## Results

### Retinal structure and vasculature remain intact in GCK^Mut^ under ND

**Figure 1:**
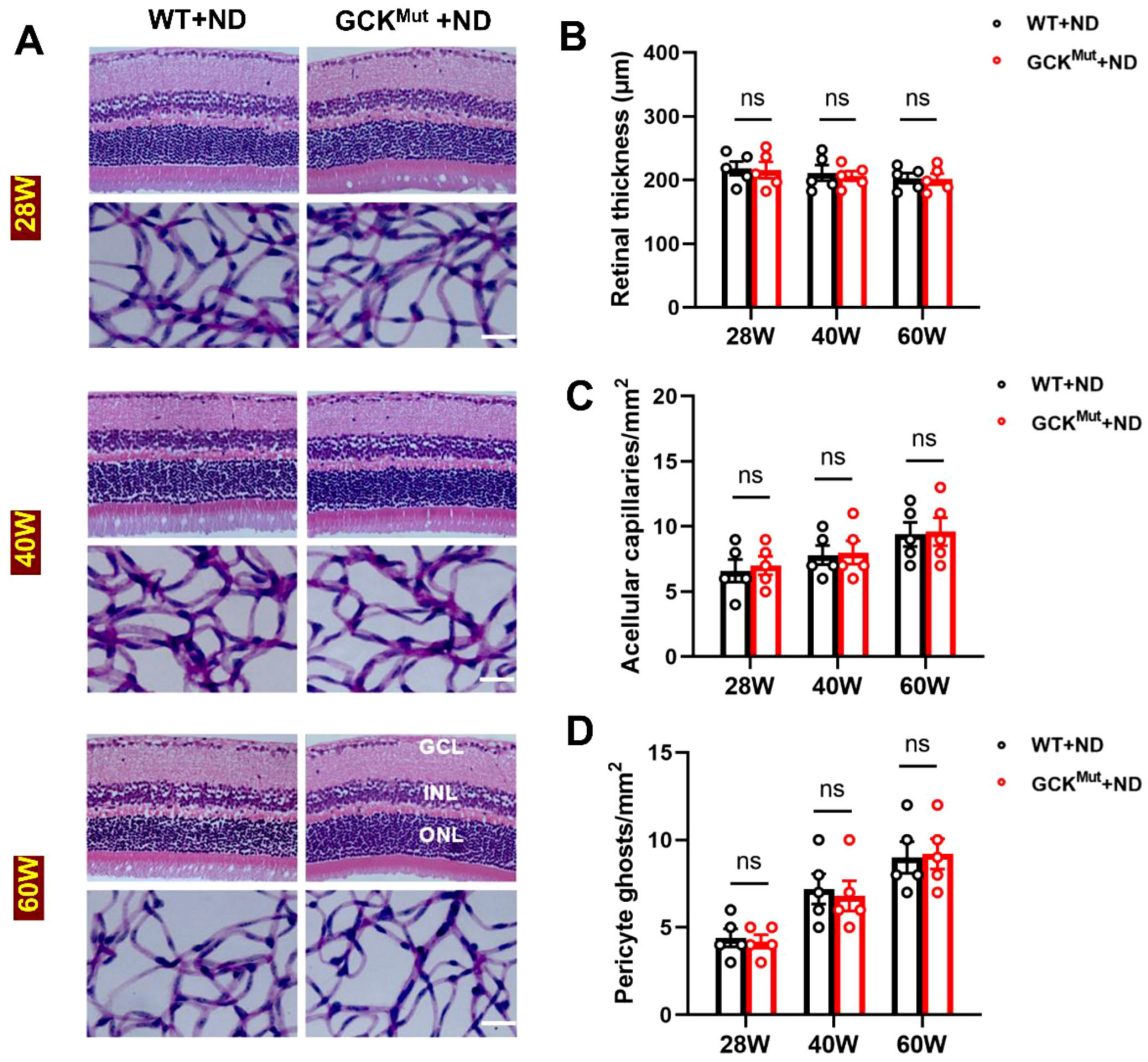
Retinal structure and microvasculature are preserved in GCK^Mut^ under ND. **(A)** Representative images of H&E staining and trypsin-digested retinal vasculature in mice from both groups at 28, 40, and 60 weeks under ND. **(B)** Quantification of total retinal thickness at different time points in each group. **(C, D)** Representative vascular images showing acellular capillaries and pericyte ghosts, and their quantification at different time points. Scale bar = 50 mm. n = 5 mice per group. Data are presented as mean ± SEM. ns: not significant (GCK^Mut^+ND vs. WT+ND). GCL, ganglion cell layer; INL, inner nuclear layer; ONL, outer nuclear layer.

Previous studies have reported significant thinning of specific retinal layers in diabetic mice (26, 27). To assess whether prolonged mild hyperglycemia affects retinal thickness, we performed H&E staining on retinal sections from mice aged 28, 40, and 60 weeks. All samples were sectioned in a consistent orientation through the optic nerve head, and measurements were obtained at a fixed distance from the nerve head to ensure data consistency. No significant differences in total retinal thickness were observed between the GCK^Mut^+ND and WT+ND groups at any examined time point (**Figures 1A, B**). Trypsin-digested retinal vascular preparations were further analyzed to evaluate retinal microvascular integrity. Consistent with the preserved retinal morphology, the numbers of acellular capillaries and pericyte ghosts were comparable between GCK^Mut^+ND and WT+ND mice at 28, 40, and 60 weeks of age (**Figures 1A, C, D**). These findings indicate that under ND conditions, GCK inactivation alone does not induce detectable retinal structural or microvascular abnormalities, even at advanced age.

### HFD induces retinal microvascular injury in aged GCK^Mut^ mice

**Figure 2:**
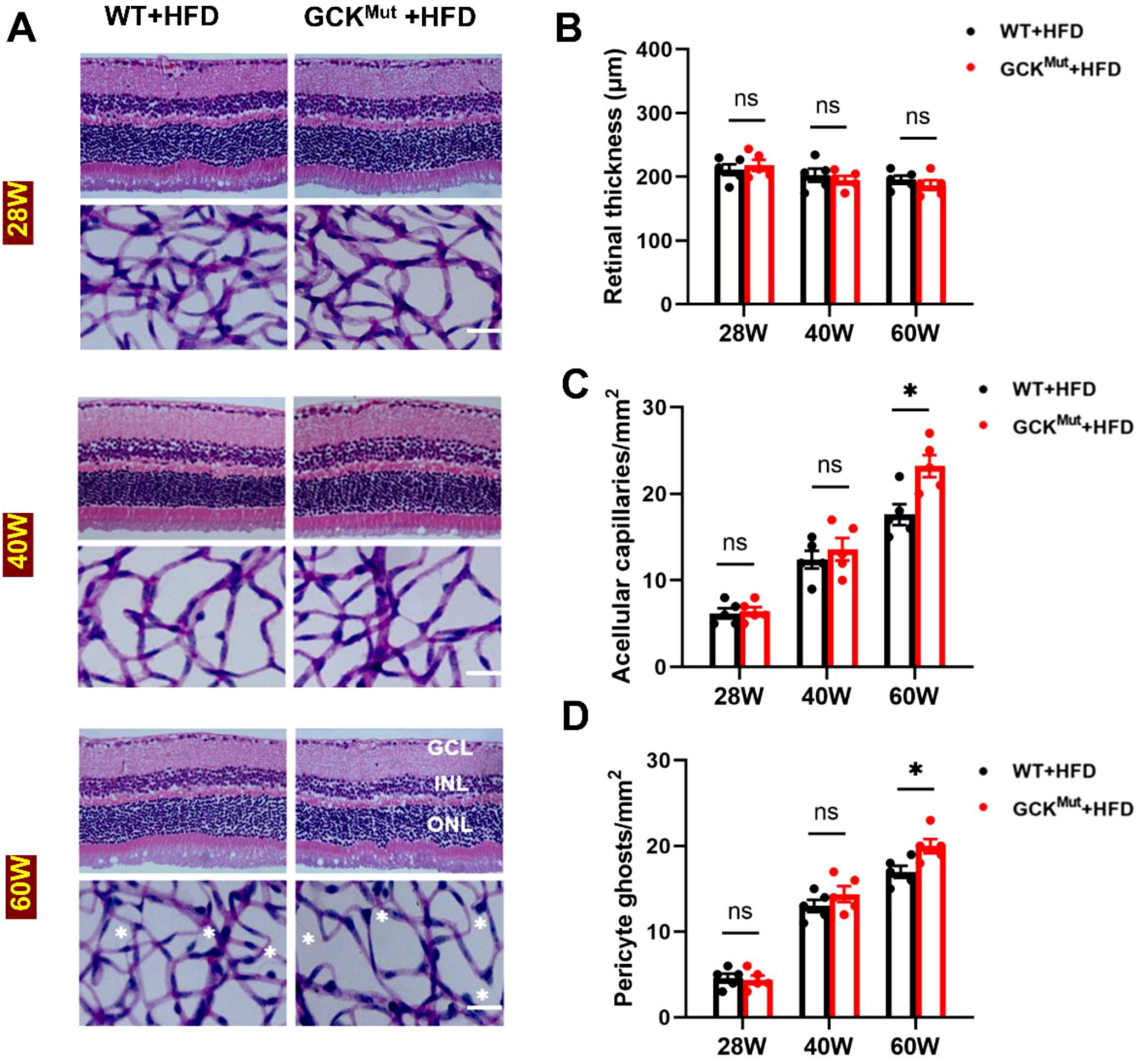
HFD induces retinal microvascular damage in aged GCK^Mut^. **(A)** Representative images of retinal morphology and vascular architecture at different time points in HFD-fed WT and GCK^Mut^ mice, assessed by H&E staining and trypsin digestion. **(B)** Quantification of total retinal thickness. **(C, D)** At 60 weeks, the GCK^Mut^+HFD group exhibited a significant increase in acellular capillaries and pericyte ghosts. Scale bar = 50 μm. n = 5 mice per group. Data are presented as mean ± SEM. *P < 0.05, ns: not significant (GCK^Mut^+HFD vs. WT+HFD). GCL, ganglion cell layer; INL, inner nuclear layer; ONL, outer nuclear layer.

In contrast to the stable retinal structure observed under ND, GCK^Mut^ mice progressively developed signs of microvascular injury under HFD. At 28 and 40 weeks, the GCK^Mut^+HFD group showed no significant differences in the number of acellular capillaries or pericyte ghosts compared with the WT+HFD group. These findings indicate that retinal microvasculature remains relatively preserved during the early stages of HFD feeding. However, at 60 weeks of age, GCK^Mut^ mice fed an HFD exhibited a marked increase in acellular capillaries and significant pericyte loss compared with age-matched WT controls. This temporal pattern demonstrates an age-dependent effect, in which retinal microvascular injury emerges only after prolonged metabolic stress (**Figures 2A, C, D**). Despite these vascular alterations, retinal thickness remained comparable between the GCK^Mut^+HFD and WT+HFD groups at all examined time points. This observation suggests that HFD-induced retinal injury in GCK^Mut^ mice is consistent with primarily microvascular involvement, with no detectable change in total retinal thickness across the examined ages (**Figures 2A, B**).

### HFD elevates retinal oxidative stress in aged GCK^Mut^ mice

**Figure 3:**
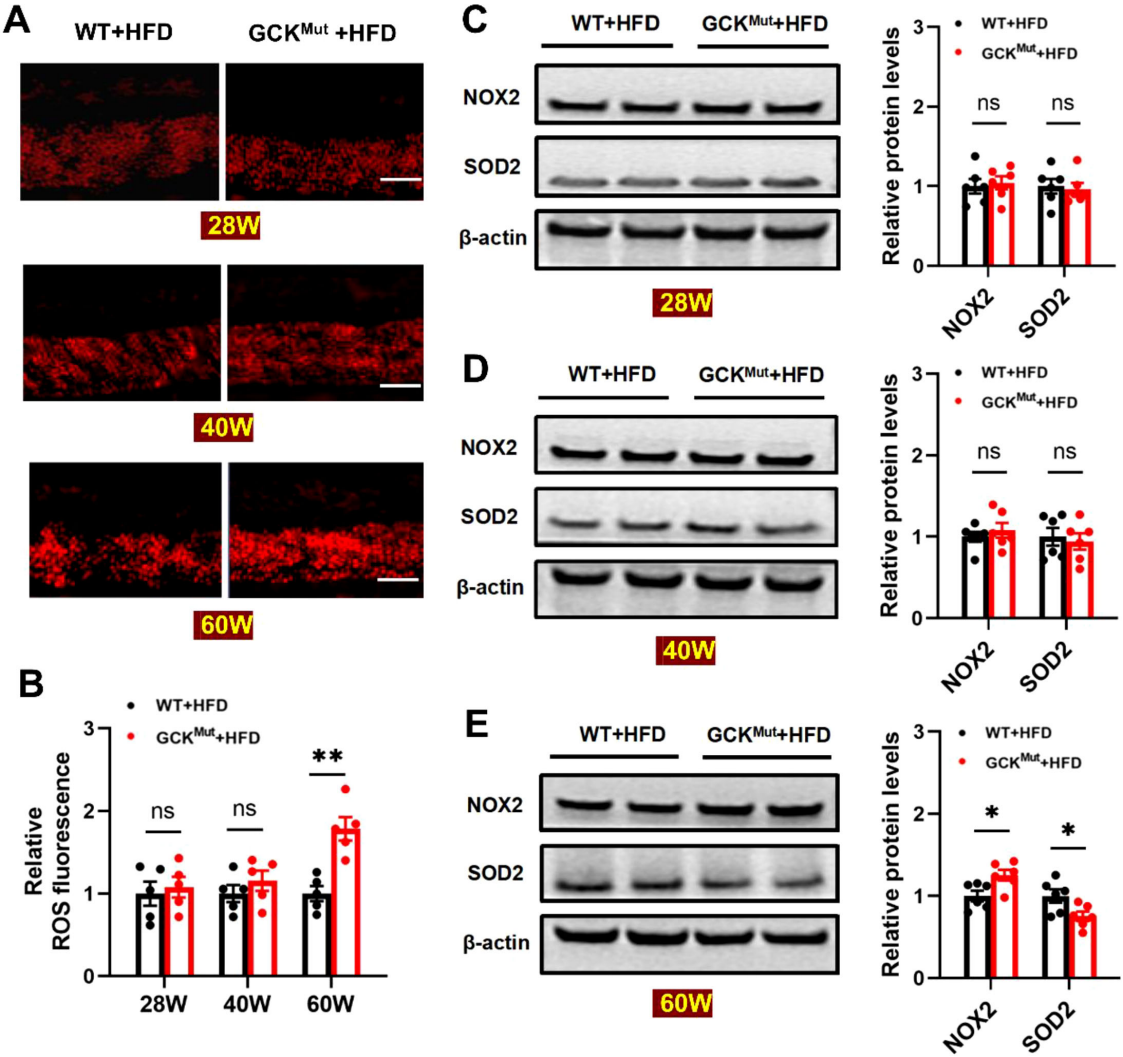
HFD increases retinal oxidative stress in GCK^Mut^ at 60 weeks. **(A)** Retinal reactive oxygen species (ROS) production at different time points was assessed by dihydroethidium (DHE) staining. **(B)** Quantification of fluorescence intensity in each experimental group. **(C–E)** Western blot analysis of NOX2 and SOD2 expression, with representative protein bands and expression levels at 28, 40, and 60 weeks of age. Scale bar = 50 μm. n = 6 mice per group. Data are presented as mean ± SEM. *P < 0.05, **P < 0.01, ns, not significant (GCK^Mut^+HFD vs. WT+HFD).

Given the pivotal role of oxidative stress in diabetic retinopathy (14–19), we further evaluated retinal oxidative stress levels in GCK^Mut^ mice under HFD. Dihydroethidium (DHE) staining showed that retinal reactive oxygen species (ROS) levels in the GCK^Mut^+HFD group were comparable to those in the WT+HFD group at 28 and 40 weeks of age, but were significantly increased at 60 weeks (**Figures 3A, B**). Analysis of oxidative stress–related proteins revealed no significant differences in NOX2 or SOD2 expression between GCK^Mut^+HFD and WT+HFD mice at 28 and 40 weeks (**Figures 3C, D**). In contrast, at 60 weeks of age, NOX2 expression was significantly upregulated, whereas SOD2 expression was markedly downregulated in GCK^Mut^+HFD mice, indicating a disruption of the oxidative–antioxidative balance (**Figure 3E**). This temporal pattern demonstrates that NOX2-associated redox imbalance emerges at the late stage of HFD exposure, coinciding with the onset of retinal microvascular injury.

To further evaluate nutrient-sensing responses under metabolic stress, we examined the activation of mTORC1 signaling in retinal tissues at 60 weeks of age. Western blot analyses of phosphorylated S6 kinase (p-S6K, Thr389) and phosphorylated S6 (p-S6, Ser240/244) showed that mTORC1 activity was comparable between WT and GCK^Mut^ mice under ND conditions. In contrast, prolonged HFD feeding induced a marked increase in mTORC1 signaling in both genotypes, with GCK^Mut^+HFD mice exhibiting a more pronounced elevation in p-S6K and p-S6 ratios compared with WT+HFD controls (**Supplementary Figure 1**). These data indicate that prolonged HFD activates retinal mTORC1 signaling, with higher p-S6K/S6K and p-S6/S6 levels in GCK^Mut^+HFD mice than in WT+HFD controls.

### Chronic HFD aggravates retinal inflammation in GCK^Mut^ mice

**Figure 4:**
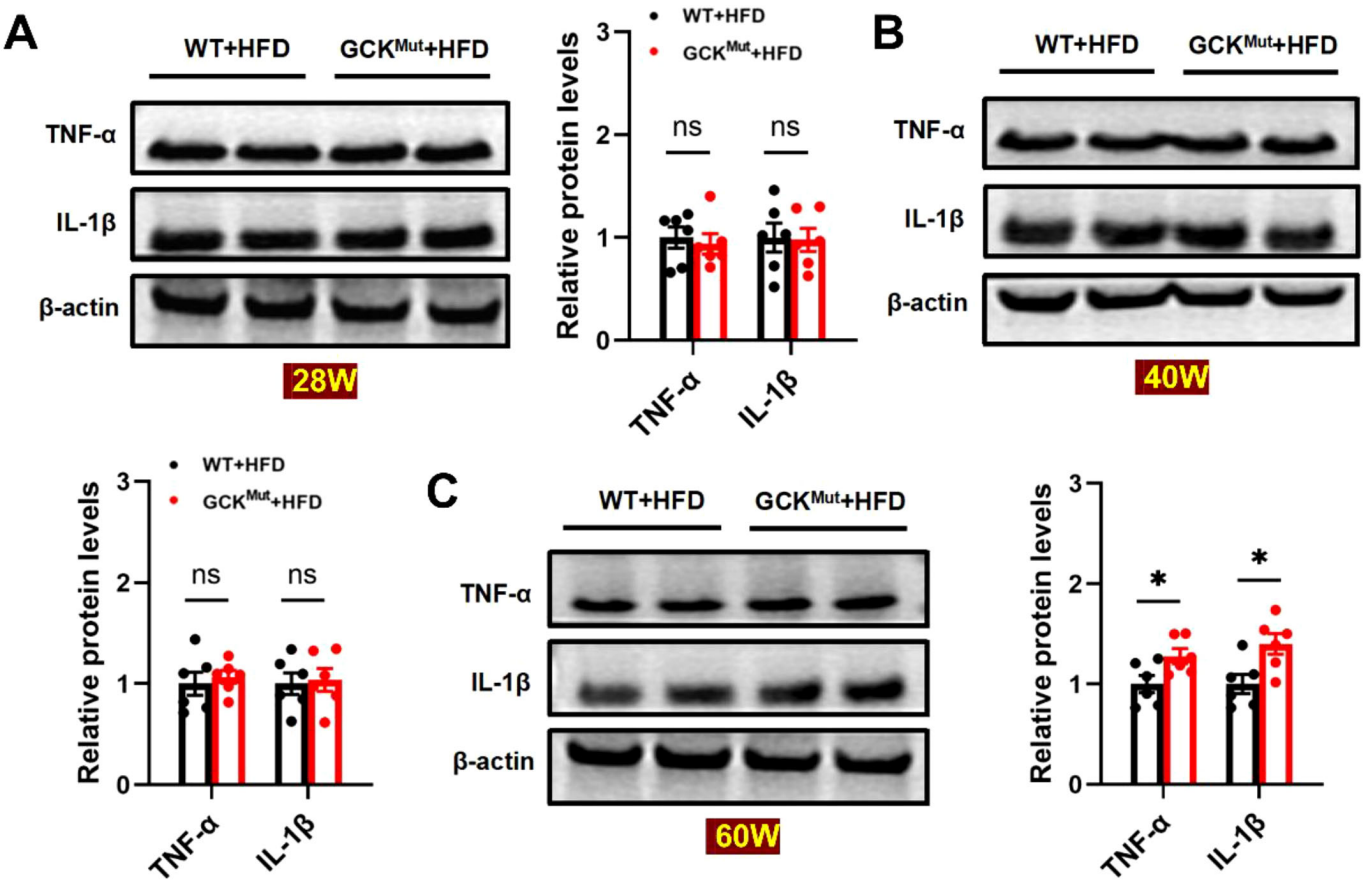
Retinal inflammation is elevated in GCK^Mut^ following prolonged HFD feeding. **(A–C)** Western blot analysis of IL-1β and TNF-α expression at 28 **(A)**, 40 **(B)**, and 60 **(C)** weeks of age, with corresponding band intensity quantification. n = 6 mice per group. Data are presented as mean ± SEM. *P < 0.05, ns: not significant (GCK^Mut^+HFD vs. WT+HFD).

Chronic inflammation is a central pathological mechanism in the development of diabetic retinopathy (28–30). To determine whether prolonged HFD feeding exacerbates retinal inflammatory responses, we examined the expression of the proinflammatory cytokines TNF-α and IL-1β in retinal tissues. At 28 and 40 weeks, TNF-α and IL-1β expression levels did not differ significantly between the GCK^Mut^+HFD and WT+HFD groups (**Figures 4A, B**). These findings indicate that retinal inflammatory activation is not evident during the early and intermediate stages of HFD exposure. In contrast, at 60 weeks of age, both TNF-α and IL-1β protein levels were significantly increased in the retinas of GCK^Mut^+HFD mice compared with WT+HFD controls (**Figure 4C**). This late-stage elevation demonstrates an age-dependent inflammatory response that parallels the emergence of microvascular injury under chronic metabolic stress. To further validate these observations at the transcriptional level, retinal inflammatory gene expression was assessed by qRT-PCR. Consistent with the protein data, Il1b and Tnf mRNA levels were significantly higher in 60-week-old GCK^Mut^+HFD mice than in WT+HFD mice (**Supplementary Figure 2**).

### HFD enhances retinal apoptosis in aged GCK^Mut^ mice

**Figure 5:**
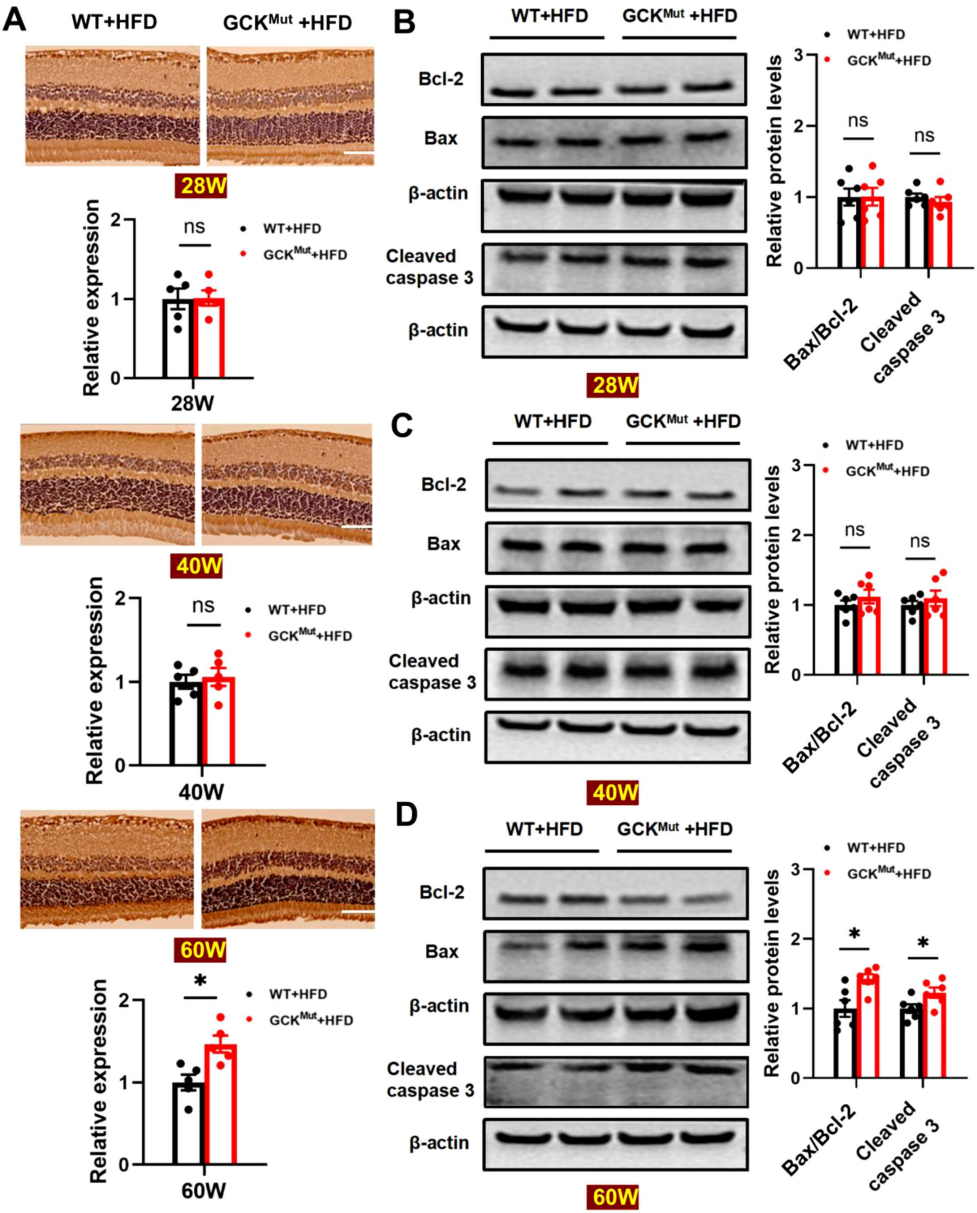
HFD feeding enhances retinal apoptosis in 60-week-old GCK^Mut^. **(A)** Representative immunohistochemical images and quantification of cleaved caspase-3 expression in both groups at 28, 40, and 60 weeks of age. **(B–D)** Western blot analysis of cleaved caspase-3, Bax, and Bcl-2 expression in both groups at all three time points, along with band intensity quantification. Scale bar = 50 μm. n = 6 mice per group. Data are presented as mean ± SEM. *P < 0.05,ns, not significant (GCK^Mut^+HFD vs. WT+HFD).

To determine whether GCK inactivation exacerbates HFD–induced retinal damage through apoptotic pathways, we evaluated the expression of key apoptosis-related markers in retinal tissues. Immunohistochemical analysis revealed that cleaved caspase-3 expression was comparable between the GCK^Mut^+HFD and WT+HFD groups at 28 and 40 weeks of age. In contrast, a marked increase in cleaved caspase-3–positive staining was observed in the retinas of 60-week-old GCK^Mut^ mice following prolonged HFD exposure (**Figure 5A**). Western blot analysis further supported these findings. At 28 and 40 weeks of age, protein levels of cleaved caspase-3, Bax, and Bcl-2 did not differ significantly between the GCK^Mut^+HFD and WT+HFD groups (**Figures 5B, C**). However, at 60 weeks of age, cleaved caspase-3 expression was significantly increased, accompanied by an elevated Bax/Bcl-2 ratio in GCK^Mut^+HFD mice compared with WT+HFD controls (**Figure 5D**). These results indicate that retinal apoptotic signaling is selectively activated at the late stage of chronic HFD exposure in the context of GCK deficiency.

### NOX2 expression correlates with inflammation and apoptosis in GCK^Mut^ retinas

**Figure 6:**
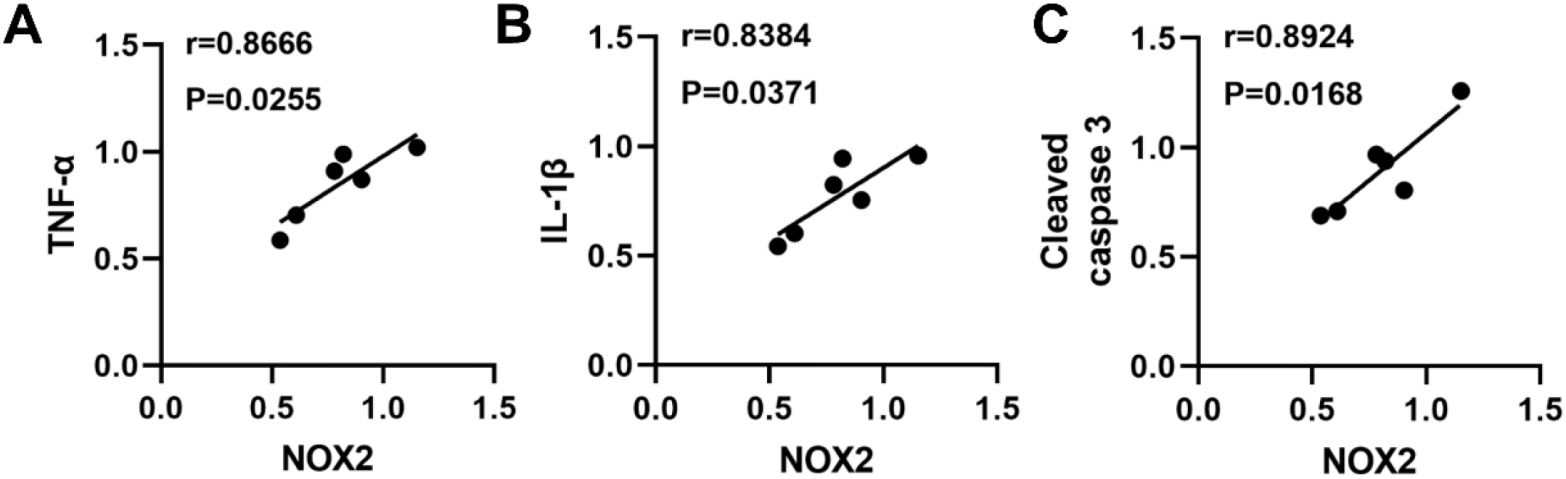
NOX2 positively correlates with inflammatory and apoptotic markers in GCK^Mut^ retinas under HFD. **(A)** Correlation between NOX2 and TNF-α: r = 0.8666, P < 0.05. **(B)** Correlation between NOX2 and IL-1β: r = 0.8384, P < 0.05. **(C)** Correlation between NOX2 and cleaved caspase-3: r = 0.8924, P < 0.05.

To explore the relationship between oxidative stress, inflammation, and apoptosis in the retinas of aged GCK^Mut^ mice under HFD conditions, linear correlation analyses were performed using retinal tissues from 60-week-old mice. NOX2 protein levels exhibited significant positive associations with the proinflammatory cytokines IL-1β and TNF-α. Specifically, NOX2 expression correlated with TNF-α (r = 0.8666, P < 0.05) and IL-1β (r = 0.8384, P < 0.05) (**Figures 6A, B**). In addition, NOX2 levels were closely associated with the apoptotic marker cleaved caspase-3 (r = 0.8924, P < 0.05) (**Figure 6C**). Together, these findings indicate that NOX2 upregulation is closely linked to inflammatory activation and apoptotic signaling in the injured retina, highlighting NOX2 as a central molecular feature associated with coordinated inflammatory and apoptotic responses in this model.

## Discussion

Using a GCK loss-of-function mouse model, we investigated retinal structural and molecular changes under chronic metabolic stress. We integrated vascular pathology with oxidative, inflammatory, and apoptotic readouts. The data support a NOX2-centered oxidative stress–inflammation–apoptosis axis that links GCK deficiency to retinal microvascular injury. This interpretation goes beyond descriptive pathology and provides a mechanistically informed framework for latent microvascular vulnerability in a traditionally mild genetic form of diabetes under sustained nutrient overload. To our knowledge, this is the first study to show that GCK inactivation can elicit retinal microvascular lesions after long-term HFD exposure, prompting a reappraisal of the benign view of GCK-MODY.

Despite marked microvascular alterations in the GCK^Mut^+HFD group at 60 weeks, total retinal thickness remained unchanged (**Figure 2**). This dissociation suggests that neurodegeneration may lag behind vascular pathology. Previous studies have similarly shown that retinal neurodegeneration and vascular remodeling are closely linked but do not progress in a fully synchronized manner (31–33). In addition, inflammatory activation and capillary occlusion can precede overt morphological degeneration (34–36). Consistent with these observations, acellular capillary formation and pericyte loss in GCK^Mut^ mice became evident only after prolonged HFD exposure at advanced age (**Figure 2**). Together, these findings indicate that retinal microvascular injury represents an early detectable, stress-dependent pathological event relative to neuroretinal thinning in the context of GCK deficiency, whereas neurodegenerative changes may require longer exposure or more severe metabolic stress to emerge.

Importantly, our findings provide evidence consistent with a NOX2-centered oxidative stress–inflammation–apoptosis axis associated with retinal microvascular injury in the setting of GCK deficiency. Microvascular lesions became evident only after prolonged HFD exposure at 60 weeks of age, indicating a clear age-dependent pattern of injury (**Figure 2**). This structural deterioration coincided with a shift toward a pro-oxidative state, characterized by increased retinal ROS and NOX2 expression together with reduced SOD2 levels (**Figure 3**). At this late stage, inflammatory cytokines were elevated at both the protein and transcript levels (**Figure 4**; **Supplementary Figure 2**), consistent with redox-sensitive inflammatory programs implicated in diabetic retinopathy (28–30). In parallel, mitochondrial apoptosis was activated, as evidenced by cleaved caspase-3 induction and an increased Bax/Bcl-2 ratio (**Figure 5**), representing a downstream consequence of sustained oxidative and inflammatory stress (20, 21). Within the same cohort, NOX2 expression showed strong associations with IL-1β, TNF-α, and cleaved caspase-3, consistent with coordinated engagement of inflammatory and apoptotic programs in the context of NOX2 upregulation (**Figure 6**).

Mechanistically, these observations are consistent with a model in which NOX2-derived reactive oxygen species may act as an upstream amplifier of redox-sensitive inflammatory signaling. Sustained NOX2 activation may also increase mitochondrial susceptibility to apoptosis, thereby promoting pericyte loss and progressive microvascular destabilization under chronic nutrient overload. This interpretation is supported by previous studies showing that NOX2 activation in retinal vascular and glial cells contributes to endothelial dysfunction, cytokine production, and capillary degeneration in diabetic retinopathy (37–40). Müller glial cells, in particular, may further propagate NOX2-driven oxidative stress and inflammatory signaling within the retinal microenvironment (41–43). Collectively, these findings position NOX2 as a molecular hub linking oxidative stress to downstream inflammatory and apoptotic injury, and highlight its potential as a therapeutic target in GCK-associated microvascular disease.

In addition to redox-driven mechanisms, nutrient- and stress-responsive signaling pathways may further modulate retinal susceptibility in the setting of GCK deficiency.

GCK is increasingly recognized not only as a glucose sensor but also as a regulator of metabolic signaling networks that coordinate cellular adaptation to nutrient availability (44). Emerging evidence suggests that GCK activity can influence pathways such as mTORC1 and inflammatory signaling programs. These pathways integrate glucose and lipid cues and regulate oxidative stress, cytokine production, and cell survival (45, 46). Consistent with this concept, our supplementary data show enhanced retinal mTORC1 activation in aged GCK^Mut^ mice following prolonged HFD exposure. This effect is reflected by increased phosphorylation of S6K and S6 (**Supplementary Figure 1**). These findings support a testable hypothesis in which chronic nutrient overload activates mTORC1 as a metabolic amplifier in GCK-deficient retinal tissue. Such activation may lower the threshold for NOX2-dependent oxidative stress and downstream inflammatory and apoptotic signaling. Future studies could directly evaluate this model by combining NOX2-targeted inhibition with mTORC1 blockade, such as rapamycin-based approaches. Assessing whether these interventions attenuate ROS accumulation, cytokine induction, and mitochondrial apoptosis in aged HFD-fed GCK^Mut^ mice would help clarify the causal hierarchy between nutrient sensing and NOX2-centered redox signaling in this context.

In contrast to our previous findings in the kidney, the retina of GCK mutant mice exhibited preserved histological structure under ND up to 60 weeks of age. In comparison, early pathological changes such as increased expression of TNF-α and IL-1β were already evident in the glomeruli at the same time point (8). This temporal divergence in microvascular pathology between the retina and kidney may reflect organ-specific differences in metabolic sensitivity and barrier integrity. The retina possesses a specialized blood–retinal barrier and a robust antioxidant defense system, which may provide greater compensatory capacity under mild hyperglycemia. In contrast, the glomerular filtration system is continuously exposed to glucose and lipid fluctuations, making it more susceptible to inflammatory activation and basement membrane remodeling. These observations underscore that GCK deficiency confers organ-specific microvascular vulnerability, with distinct temporal windows for injury manifestation under metabolic stress.

Although this study systematically elucidates mechanisms underlying retinal microvascular injury in GCK-inactivated mice under HFD conditions, several limitations warrant consideration. First, the GCK-Q26L knock-in model represents a single mutation and does not capture the full mutational and phenotypic heterogeneity of human GCK-MODY. Second, mechanistic inference is based primarily on tissue-level associations, and causal validation of the proposed NOX2-centered pathway will require targeted genetic or pharmacological interventions. Third, only male mice were included, precluding assessment of sex-specific effects. Fourth, translational relevance remains to be established in clinical cohorts. Future studies combining NOX2 inhibition with modulation of nutrient-sensing pathways such as mTORC1 will be particularly informative in testing the proposed mechanistic framework.

In summary, this study delineates the age-dependent progression of retinal microvascular damage induced by long-term HFD exposure in a GCK loss-of-function mouse model. Our findings suggest that NOX2 contributes to retinal pathogenesis under chronic metabolic stress. These results indicate that GCK-MODY may harbor a latent risk of microvascular disease progression when exposed to common stressors such as HFD, obesity, or aging. Collectively, this work warrants a re-evaluation of the complication risk spectrum in GCK-MODY and provides a rationale for more proactive, mechanism-informed management strategies.

## Data Availability

The raw data supporting the conclusions of this article will be made available by the authors, without undue reservation.
