# Supplementary material for: Age- and diet-dependent progression of retinal microvascular injury in GCK-MODY under metabolic stress
Source: Front Endocrinol (Lausanne). 2026 Mar 6;17:1744691. doi: 10.3389/fendo.2026.1744691 (PMC13002402; doi:10.3389/fendo.2026.1744691)
Supplement: Supplementary file 1 [file DataSheet1.docx]

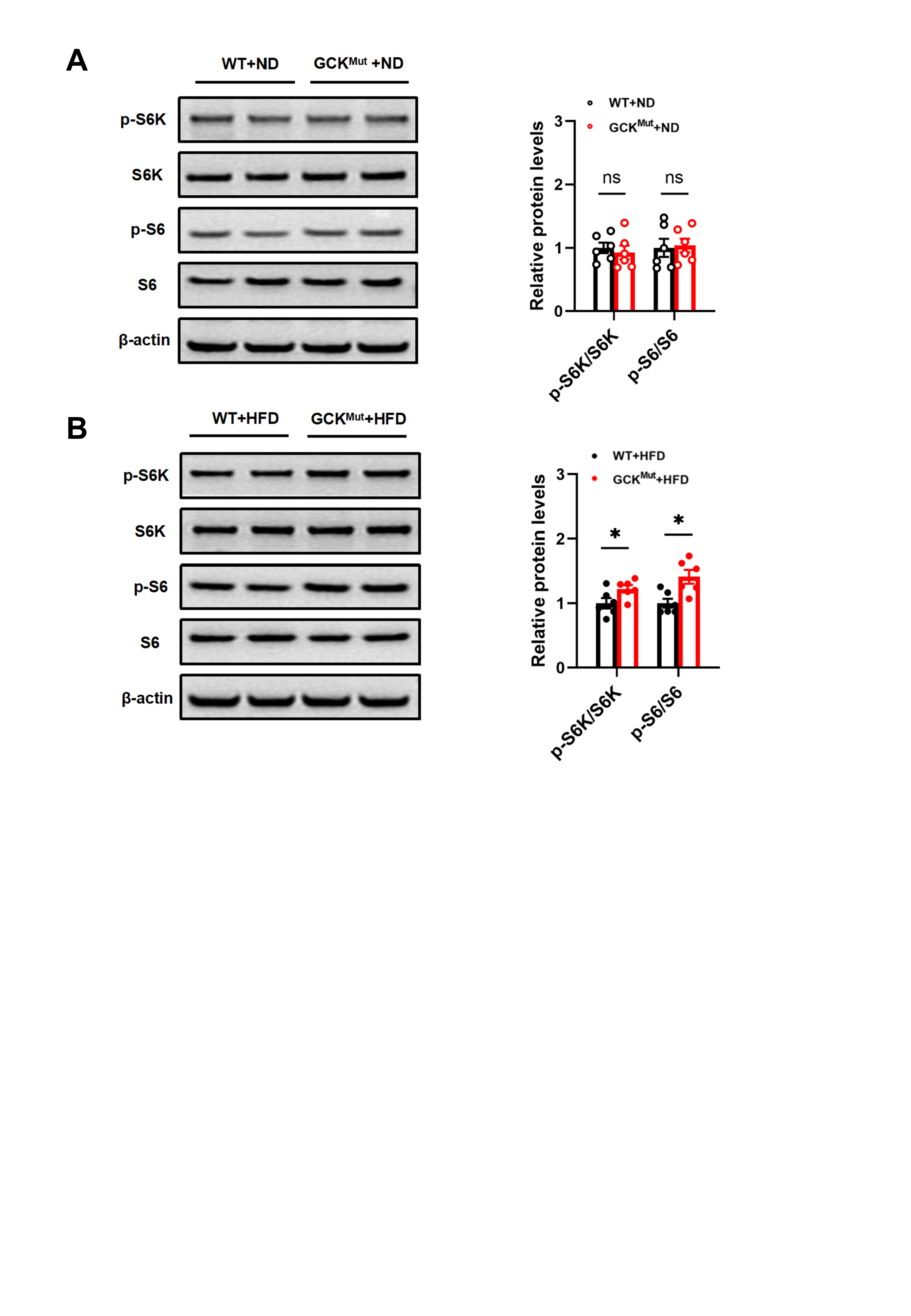


**Supplementary Figure 1. Retinal mTORC1 signaling in WT and GCK^Mut^ mice at 60 weeks under ND and HFD.** (A) Representative Western blots and densitometric quantification of p-S6K/S6K and p-S6/S6 in retinas from WT+ND and GCK^Mut^+ND mice at 60 weeks of age. (B) Representative Western blots and densitometric quantification of p-S6K/S6K and p-S6/S6 in retinas from WT+HFD and GCK^Mut^+HFD mice at 60 weeks of age. n = 6 mice per group. Data are presented as mean ± SEM. *P < 0.05, **P < 0.01, ns: not significant (GCK^Mut^ vs. WT within each diet condition).


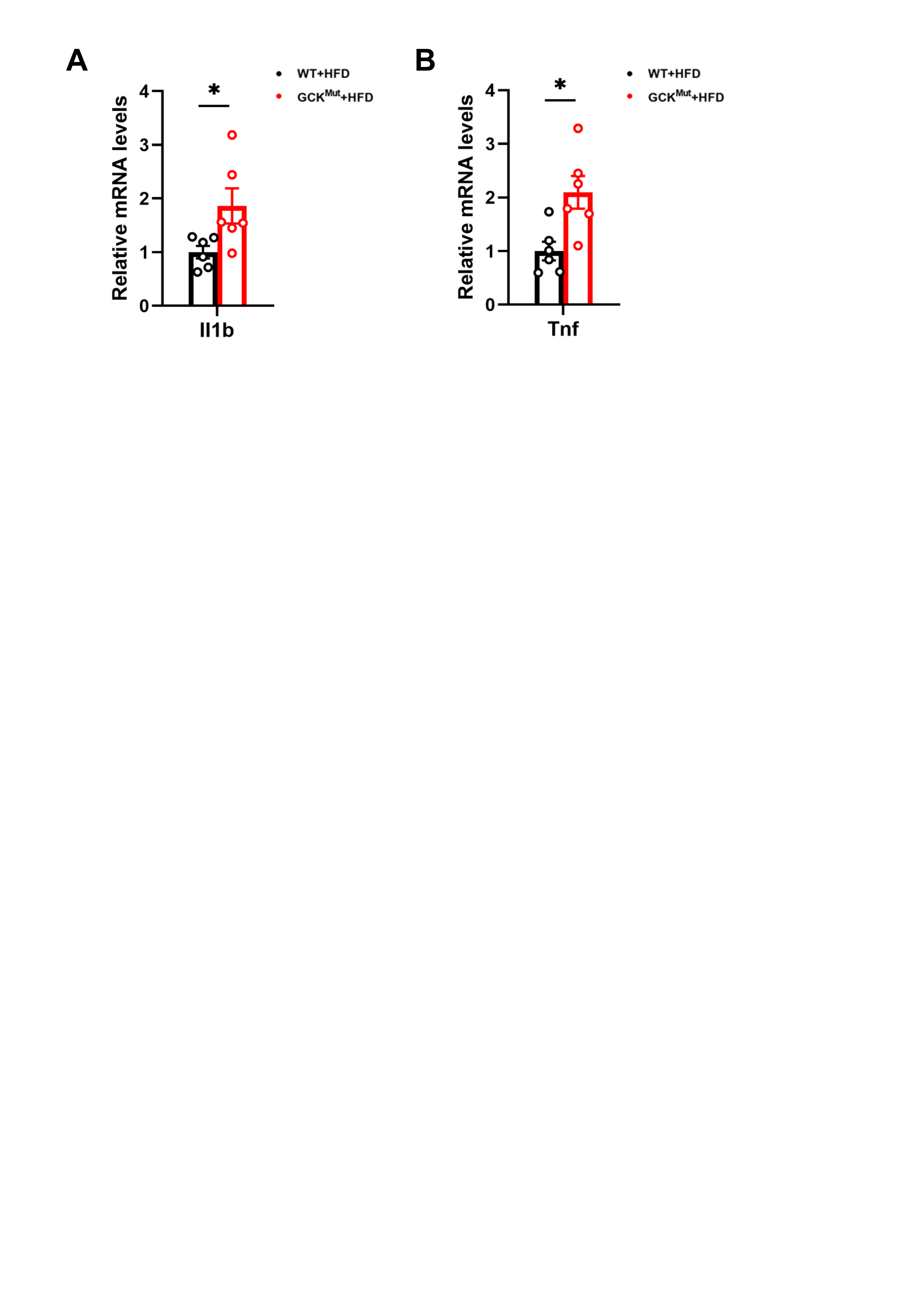


**Supplementary Figure 2. Quantitative real-time PCR analysis of retinal inflammatory cytokines in 60-week-old mice subjected to prolonged HFD.** Relative mRNA expression levels of Il1b (A) and Tnf (B) were measured in WT+HFD and GCK^Mut^+HFD retinas. n = 6 mice per group. Data are presented as mean ± SEM. *P < 0.05, **P < 0.01, ns: not significant (GCK^Mut^+HFD vs. WT+HFD).

**Supplementary Table 1. Primer sequences (m-Mus musculus) for qRT-PCR (5’-3’)**

| Gene | Forward | Reverse |
| --- | --- | --- |
| mTNF-α | GCGACGTGGAACTGGCAGAAGAGG | GTACTTGGGCAGATTGACCTCAGC |
| mIL-1β | CACTACAGGCTCCGAGATGAACAAC | TGTCGTTGCTTGGTTCTCCTTGTAC |
| mGAPDH | ACCCACTCCTCCACCTTTGAC | TGTTGCTGTAGCCAAATTCGTT |
